# *In vitro* electrophysiological characterization of Parkinson’s disease: challenges, advances, and future directions

**DOI:** 10.3389/fnins.2025.1584555

**Published:** 2025-04-30

**Authors:** Sara Grasselli, Andrea Andolfi, Donatella Di Lisa, Laura Pastorino

**Affiliations:** ^1^Department of Informatics, Bioengineering, Robotics and Systems Engineering (DIBRIS), University of Genoa, Genoa, Italy; ^2^IRCCS Ospedale Policlinico San Martino, Genoa, Italy

**Keywords:** Parkinson’s disease, electrophysiology, *in vitro* models, patch-clamp, microelectrode arrays

## Abstract

Parkinson’s disease is the second most common neurodegenerative disorder, characterized by the progressive loss of dopaminergic neurons in the substantia nigra and the accumulation of *α*-synuclein aggregates. While significant progress has been made in understanding the genetic and biological aspects of Parkinson’s disease, its complex pathophysiology remains poorly understood, and current therapeutic approaches are largely symptomatic. Advanced *in vitro* models have emerged as essential tools for studying Parkinson’s disease related mechanisms and developing new therapeutic strategies. However, the electrophysiological characterization of neurons in these models remains underexplored. This review highlights the importance of employing electrophysiological techniques, such as patch-clamp recordings and microelectrode arrays, in providing critical insights into neuronal dysfunction, synaptic impairments, and network disruptions in Parkinson’s disease. The aim is to summarize the key discoveries in the electrophysiological characterization of the pathology and the related progress made in recent years, underlying the main challenges, including the lack of standardized protocols, and the heterogeneity of cellular sources and culture systems. Addressing these limitations is crucial for improving reproducibility and facilitating cross-study comparisons, allowing for a deeper understanding of Parkinson’s disease pathophysiology. By refining and standardizing electrophysiological approaches, these efforts will enhance our understanding of Parkinson’s disease’s underlying mechanisms, ultimately accelerating the discovery of robust biomarkers and the development of more effective therapeutic strategies.

## Introduction

Parkinson’s disease (PD) is the second most common neurodegenerative disorder, affecting approximately 3% of the population over the age of 65 (Armstrong and Okun, 2020; Tysnes and Storstein, 2017).

Clinically, PD is characterized not only by features such as tremor, rigidity, and bradykinesia, but also a spectrum of non-motor manifestations that complicate its clinical progression (Hayes, 2019; Jankovic, 2008). These multifaceted symptoms impair the quality of life (Fasano et al., 2015) and underscore the pressing need for comprehensive research into its underlying mechanisms. Moreover, the increasing life expectancy is contributing to a higher incidence of neurodegenerative conditions, further emphasizing the critical need for effective therapeutic interventions (Abdullah et al., 2015; Hou et al., 2019; Poewe et al., 2017).

At the molecular level, PD is linked to genetic mutations associated with *α*-synuclein (αSyn) aggregation in neurons (Atik et al., 2016; Chen et al., 2022; Dong-Chen et al., 2023; Rocha et al., 2018). This leads to dopaminergic neuron (DNs) degeneration in the substantia nigra (Kamath et al., 2022; Marogianni et al., 2020) causing dopamine deficiency and movement impairments (Bloem et al., 2021; Jankovic and Tan, 2020; Morris et al., 2024).

To date, L-DOPA administration (de Bie et al., 2020; Murakami et al., 2023; Pirker et al., 2023) and deep brain stimulation (DBS) represent the most effective treatments for managing PD symptoms (Beudel and Brown, 2016; Gao et al., 2020; Hariz and Blomstedt, 2022). However, these approaches are non-curative, with L-DOPA use often leading to long-term complications such as dyskinesias and motor fluctuations (Angela Cenci, 2014; Olanow et al., 2006; Poewe et al., 2017), and DBS requiring invasive brain surgery with associated operative and post-operative risks (Voges et al., 2007; Volkmann et al., 2010). Other pharmacological options provide additional symptom relief but are often limited by side effects (Cattaneo and Jost, 2023; Poewe et al., 2017; Schindler et al., 2021). Despite these advances, the urgent need for refined early identification of PD and the development of novel, curative therapies remains.

The development of *in vitro* models has improved our ability to investigate the molecular and functional basis of PD. Techniques utilizing stem cells (Kamath et al., 2022b; Nakamura et al., 2023), organoids (Wulansari et al., 2021; Zheng et al., 2023), and cultured neural networks have emerged as invaluable tools, replicating key aspects of the disease and providing platforms for both mechanistic studies and therapeutic screening. These models allow researchers to dissect complex disease processes under controlled conditions, bridging the gap between molecular pathology and clinical presentation. Investigating the electrophysiological mechanisms underlying PD pathogenesis is essential for ameliorating our understanding of the pathology and for identifying new potential therapeutic targets (Branch et al., 2016; Tønnesen et al., 2011; Zhu et al., 2002). Increasing evidence suggests a close link between electrophysiological impairments and certain PD-related mutations. This indicates that these mutations may contribute to disease symptoms and progression, highlighting the complex interplay between genetic factors and neuronal function.

**Figure 1:**
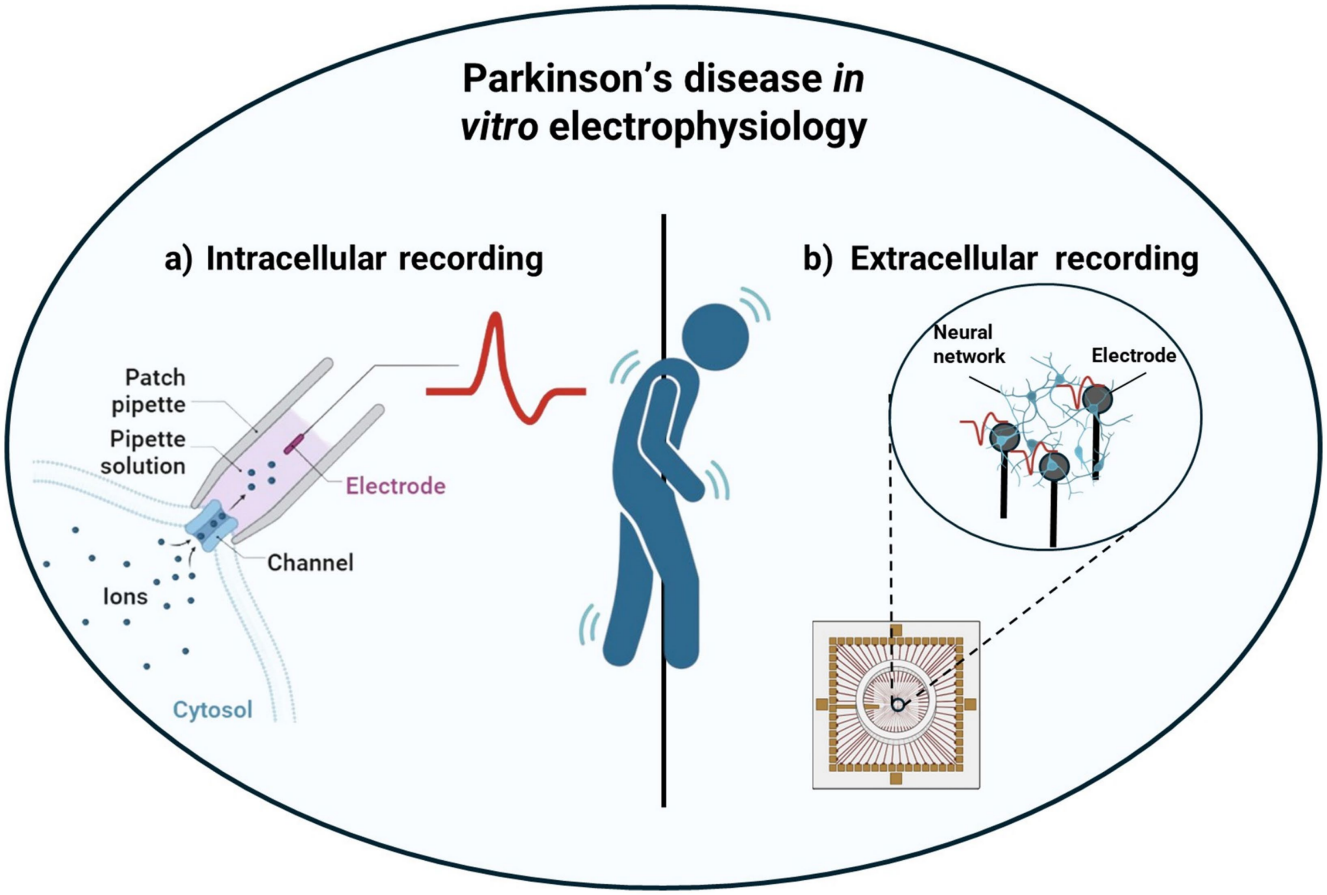
Schematical representation of Parkinson’s disease characterization leveraging *in vitro* electrophysiological techniques: from intracellular recordings using patch-clamp **(a)** to extracellular recordings using MEAs **(b)**.

However, relatively few studies have employed electrophysiological techniques to gain novel insights into the disease and substantial gaps remain in understanding the electrophysiological dynamics of PD models. Addressing these gaps is pivotal for the development of innovative therapeutic strategies and the creation of reliable predictive models for disease progression. This review aims to underscore the importance of an electrophysiological approach in analyzing neural networks affected by Parkinson’s-related mutations. It presents key findings from recent studies employing patch-clamp recordings (Figure 1a) and microelectrode arrays (MEAs; Figure 1b), while also addressing current challenges and future research directions in the field.

## Investigation of intracellular and extracellular neural activity

Patch-clamp is a highly sensitive electrophysiological technique for studying ionic currents and membrane potentials in electrogenic cells (Hamill et al., 1981; Hill and Stephens, 2021). It can be applied to isolated cell cultures (Rosholm et al., 2021; Takasuna et al., 2017; Toh et al., 2020) or tissue slices (Segev et al., 2016; Ting et al., 2014), offering precise control over the cellular environment and enabling high-resolution recordings of electrical activity. Patch-clamp experiments can be conducted in voltage-clamp or current-clamp modes (Ghovanloo et al., 2023), where the voltage or current across the cell membrane is controlled, respectively. One of its major advantages is the ability to access the intracellular environment directly, as well as to perform whole-cell measurements with exceptional spatiotemporal precision. Due to these advantages, patch-clamp has been extensively used to explore key electrophysiological mechanisms in both physiological and pathological contexts. In PD research, it serves as a crucial tool for studying the disease’s electrophysiological manifestations *in vitro*.

A pivotal study by Chan et al. (2011) demonstrated that dopamine (DA) depletion in rodent PD models downregulates hyperpolarization-activated cyclic nucleotide-gated (HCN) channels in neurons of the external globus pallidus (GPe), leading to a loss of autonomous pacemaking activity. Additionally, viral restoration of HNC channels by HNC2 delivery resulted in the recovery of spontaneous activity of neurons of the GPe, while motor disability caused by DA depletion was not resolved, suggesting that the decrease in neuronal activity is a consequence of PD progression and not a cause. These findings contribute to broader efforts aimed at understanding how PD-associated pathological changes disrupt neuronal excitability. Further emphasizing the importance of cellular models in PD research, Ross et al. (2020) utilized the SH-SY5Y cell line as an *in vitro* PD model, showing that the accumulation of αSyn fibrils within cell bodies increases the action potential threshold, significantly impacting neuronal excitability. Interestingly, they demonstrated how inducing membrane depolarization, increasing the extracellular concentration of K^+^ or blocking the GABA receptor significantly reduced the cytotoxic effect of αSyn aggregation, thus providing a potential therapeutic target. A similar effect was witnessed by direct current stimulation, a clinically used treatment for reducing αSyn toxicity.

More recent advancements have leveraged hiPSCs to create patient-specific models, enabling clinically relevant insights into PD. Rakovic et al. (2022) employed hiPSCs-derived midbrain DNs (mDAs) to model PD, comparing the electrophysiological properties of tyrosine hydroxylase-positive (TH+) and -negative cells (TH-). Their findings suggest that TH + neurons exhibit unique electrophysiological properties that may contribute to their selective vulnerability in PD.

Other studies have combined hiPSCs with patch-clamp recordings to explore PD-related mutations. Virdi et al. (2022) conducted whole-cell voltage- and current-clamp recordings on hiPSC-derived DNs carrying the SNCA mutation, revealing impaired electrophysiological properties compared to isogenic controls. They demonstrated how αSyn aggregation and calcium dysregulation are the first manifestations of PD progression in neurons carrying the SNCA mutation, followed by mitochondrial dysfunction and increased oxidative stress that led to impaired neuronal excitability. Similarly, Do et al. (2024) demonstrated that hiPSC-derived DNs with the GBA-N370S mutation caused early hyperexcitability in medium spiny neurons (MSNs), suggesting potential electrophysiological biomarkers for PD. The microfluidic platform developed in this study allowed the precise control of neuronal connectivity between DNs and MSNs, thus providing a suitable model for the investigation of specific neuronal interactions.

Expanding on this approach, Smits et al. (2019) developed 3D midbrain organoids from DNs derived from healthy donors and PD patients carrying the LRRK2-G2019S mutation. They demonstrated how patch-clamp can be used to record electrophysiological signals from 3D organoids, providing a more faithful model than traditional 2D cell cultures, making them valuable alternatives for further disease characterization.

Overall, patch-clamp techniques provide precise recordings of electrical potentials and sub-threshold signals in single neurons but are invasive, labor-intensive, and unsuitable for prolonged studies due to reduced cell viability.

Compared to patch-clamp, MEAs allow non-invasive, multi-site recordings of extracellular field potentials and spike activity with high spatiotemporal resolution, thus enabling long-term observation of neural networks and offering unique insights into their developmental dynamics (Obien et al., 2015; Spira and Hai, 2013). These advantages make MEA recordings particularly valuable for disease modeling, drug screening, and *in vitro* studies of complex neural processes. Consequently, MEAs have been extensively used to enhance the understanding of neurodegenerative disorders (Amin et al., 2017; O’Connell et al., 2024), including PD.

In this context, Reumann et al. (2023) developed a novel *in vitro* model based on Midbrain-Striatum-Cortex Organoids (MISCOs), which replicates key features of the dopaminergic system, including striatal and cortical innervation. By spatially integrating ventral midbrain, striatal, and cortical organoids, MISCOs establish functional long-range dopaminergic connections, providing a platform for studying dopaminergic neuron development, maturation, and degeneration in PD research. MEA probe analysis confirmed the functional connectivity of MISCOs. Additionally, calcium imaging detected spontaneous intracellular calcium transients in all three regions, while dopaminergic axons exhibited calcium events in forebrain areas, indicating long-range connectivity. These findings demonstrate MISCOs’ ability to model PD-related connectivity loss and evaluate potential therapies, particularly for connectivity restoration. Despite limitations such as the absence of vascularization and incomplete maturation, MISCOs represent a promising tool for investigating network dysfunctions and restorative interventions in PD.

The study by Lin et al. (2016) provides a deeper understanding of the molecular and functional disruptions in PD, using hiPSCs-derived mDAs from patients carrying different PD-related mutations to identify key molecular features of neurodegeneration. Their goal was to unravel the common downstream signaling pathways driving PD progression across various genetic predispositions. Electrophysiological experiments using a 256-channel MEAs revealed that while both wild-type (WT) and PD-derived mDAs exhibited spontaneous action potentials, only WT neurons displayed synchronized network firing. In contrast, PD-derived neurons, particularly those with the LRRK2-G2019S mutation, lacked synchronized activity, highlighting impaired neural connectivity, a hallmark of PD pathology. Moreover, PD neurons demonstrated significantly reduced firing rates and active channels compared to WT, suggesting diminished neuronal excitability and network function. These deficits were further validated through pharmacological experiments showing functional NMDA and dopamine D2 receptor responses in both WT and PD neurons, confirming their physiological relevance. The electrophysiological experiments indicated that PD-derived mDA neurons exhibit significant deficits in neuronal activity and network synchronization, suggesting that PD neurons have compromised functional properties, which could reflect the neurodegenerative processes seen in PD.

Laperle et al. (2020) gave a significant contribution to young-onset Parkinson’s disease (YOPD), a condition accounting for around 10% of PD cases and typically emerging before age 50. Given that over 80% of YOPD cases lack a family history or identifiable genetic mutations, the study aimed to uncover its molecular mechanisms. To identify disease-specific phenotypes and potential therapeutic targets, the researchers differentiated hiPSCs from YOPD patients and healthy controls into mDAs. Employing patch-clamp techniques, they observed that YOPD neurons displayed impaired excitability, characterized by a reduced sodium current amplitude. This deficit was likely attributable to elevated phosphorylated PKCα levels, a negative regulator of sodium channels, indicating broader disruptions in PKC and PKA signaling pathways. MEA recordings further revealed a reduction in spike frequency, highlighting a decrease in neuronal activity and network connectivity, both crucial for dopaminergic function. These findings support the broader hypothesis that YOPD neurons experience diminished functional capabilities contributing to neurodegeneration.

Expanding on the role of genetic and epigenetic influences in PD, Woodard et al. (2014) investigated monozygotic twins discordant for the disease, analyzing mDAs derived from hiPSCs. Through a combination of MEA and patch-clamp recordings, they identified impaired network activity in neurons from the affected twin, marked by reduced spontaneous firing and an absence of synchronized bursting, indicative of delayed or disrupted network maturation. Patch-clamp analyses further confirmed decreased excitability in these neurons. Interestingly, despite carrying the same GBA-N370S mutation, the unaffected twin’s neurons exhibited normal function, emphasizing the potential role of epigenetic and environmental factors in PD progression.

Further exploring αSyn pathology, Valderhaug et al. (2021) examined its impact on network function using hiPSC-derived neuronal networks. By introducing preformed fibrils (PFFs) to model PD pathology, they conducted MEA recordings to assess network activity. While traditional metrics such as mean firing rate and cross-correlation did not reveal significant differences between PFF-treated and control networks, criticality analysis suggested a greater tendency toward self-organized criticality (SoC) in PFF-treated networks, potentially as an adaptive response to pathology. These findings highlight SoC as a sensitive tool for detecting early network alterations in PD, providing new insights into neurodegenerative disease progression.

Building on this, Ronchi et al. (2021) investigated the electrophysiological properties of human dopaminergic neurons (hDNs) and their isogenic counterparts carrying the A53T αSyn mutation (hDN-PD), linked to PD. MEA recordings revealed that hDN-PD neurons exhibited a reduced firing rate but an increased burst frequency with shorter, more variable inter-burst intervals, indicating impaired network synchronization. Despite these network irregularities, axonal propagation velocity remained unaffected, suggesting that PD-related dysfunction arises primarily from intrinsic firing and synaptic alterations rather than conduction deficits. By pinpointing specific electrophysiological disruptions, this study offers valuable insights into PD pathology and potential therapeutic targets.

Due to their distinct methodologies, patch-clamp and MEA techniques offer complementary insights into the electrical activity of Parkinsonian neural networks. Table 1 provides a comparative summary of their respective advantages and shortcomings.

**Table 1:** Comparison of features and performance of patch-clamp and MEA techniques for the electrophysiological characterization of *in vitro* neural cultures.

Feature	Patch clamp	Microelectrode array (MEA)
Principle	Measures ion currents through individual ion channels using a glass micropipette	Measures extracellular field potentials from multiple cells using an array of electrodes
Resolution	High-resolution, single-channel, membrane potentials, and synaptic events recording	Lower resolution, single-cell or population-level activity recording
Throughput	Low (recordings from one cell at a time)	High (simultaneous recording from multiple cells)
Invasiveness	Invasive; requires membrane penetration or tight-seal formation	Non-invasive; cell adhere and grow on the electrodes
Suitability for long-term studies	Limited due to potential cell damage	Suitable for long-term monitoring of neuronal networks (up to several weeks)
Data complexity	Precise, detailed data on ion channel kinetics	Provides broader, less detailed information on network activity
Ease of use	Technically demanding; requires expertise	Easier to use once electrodes are placed
Cost	Expensive equipment	Moderate to high

Considering their unique characteristics, patch-clamp and MEA techniques are complementary rather than mutually exclusive (Jäckel et al., 2017).

Patch-clamp excels in detailed investigations of ion channel dysfunction (Bednarczyk et al., 2021; Toh et al., 2020), synaptic abnormalities, and neuronal excitability in PD models, for example, examining the effects of αSyn aggregation on membrane potential (Ross et al., 2020). In contrast, MEA is particularly valuable for analyzing network dysfunction, abnormal synchronization, and large-scale neuronal activity changes observed in PD, such as the loss of connectivity in dopaminergic circuits (Reumann et al., 2023; Spira and Hai, 2013).

In summary, patch-clamp is ideal for mechanistic studies at the cellular level, while MEA is better suited for capturing broader network dysfunctions over time.

## Discussion

Despite substantial progress in understanding its clinical manifestations (Tysnes and Storstein, 2017), the complex pathophysiological mechanisms driving PD progression remain incompletely understood. Animal models have traditionally played a central role in PD research (Sturchio et al., 2024; Zeiss et al., 2017), but they face significant ethical, economical, and translational limitations (Barré-Sinoussi and Montagutelli, 2015). Conversely, *in vitro* models offer a more controlled environment for studying key cellular processes and have become essential tools for investigating disease mechanisms and identifying therapeutic targets (Travagli et al., 2020; Vargas et al., 2019).

*In vitro* models for electrophysiological studies have played a crucial role in revealing key disruptions in neuronal excitability, synaptic dynamics, and network synchronization in PD (Kouroupi et al., 2017; Negri et al., 2020; Puopolo et al., 2007). Studies using patch-clamp and MEA techniques have identified abnormal firing patterns, altered pacemaking activity, and deficits in coordinated network behavior in DNs.

Recent advances in 3D culture systems, such as spheroids, scaffold-based constructs, and organoids, have further transformed PD modeling by better recapitulating the spatial complexity and microenvironment of neural tissue (Abdelrahman et al., 2022). Notably, hiPSC-derived neurons and 3D organoid models have enabled researchers to link specific genetic mutations with distinct electrophysiological phenotypes (Kouroupi et al., 2020). Despite these achievements, the current body of research leaves several critical areas insufficiently explored.

One emerging challenge is the need to capture long-term electrophysiological adaptations in *in vitro* models (Monchy et al., 2024; Xu et al., 2023). While current techniques effectively reveal acute changes in neuronal behavior, chronic adaptations such as synaptic remodeling, homeostatic plasticity, and compensatory mechanisms remain poorly understood. Investigating these processes is essential for understanding the progressive nature of PD, particularly in the context of gradual DNs degeneration and its impact on network-wide properties (Rassoulou et al., 2024). In parallel, the mechanisms underlying pathological neuronal synchronization, especially within the beta frequency range, a hallmark of PD, are not yet fully understood. Investigating how intrinsic neuronal properties and synaptic interactions drive these abnormal oscillations in controlled *in vitro* settings could yield valuable insights into disease pathophysiology (Viegas et al., 2023).

Adding further complexity, the integration of multi-scale electrophysiological techniques with calcium imaging has provided a more comprehensive perspective on PD-related dysfunction (Beccano-Kelly et al., 2023). For example, studies have demonstrated that knocking down Homer1 in DNs significantly reduces calcium overload following MPP^+^-induced injury, highlighting calcium dysregulation as a critical factor in PD pathology (T. Chen et al., 2013). Similarly, research using hiPSC-derived PD models carrying the p.A53T mutation has revealed increased spontaneous calcium transients associated with morphological and synaptic deficits, suggesting that early disruptions in calcium signaling may contribute to disease onset (Zygogianni et al., 2019). This integrative approach, which links subcellular events with broader network interactions, underscores the importance of multi-modal investigations in developing a complete picture of neuronal dysfunction in PD. In this regard, a compelling example integrating MEA with imaging explores the influence of specific factors on Parkinson’s disease, focusing on neural activity and potential biomarkers (Ozgun et al., 2024). To achieve this, the study utilized calcium imaging to demonstrate spiking synchronicity between organoids, confirming functional connectivity. MEA recordings validated a robust response to the dopamine neurotoxin 6-OHDA, highlighting the potential of this technology to study PD’s dysfunctional dopaminergic network. Through these methodologies, the study identifies critical differences in neuronal behavior associated with Parkinson’s, potentially revealing biomarkers or therapeutic targets for improved diagnosis and treatment strategies.

Complementing these technical advancements, the dynamic modulation of dopaminergic tone by neuromodulators offers another promising avenue of research. Although *in vitro* systems provide the controlled environments necessary for systematic pharmacological manipulations, a deeper exploration into how these neuromodulatory influences affect both the intrinsic properties of dopaminergic neurons and their synaptic connectivity is essential (Cole et al., 2022). Such studies will refine our understanding of therapeutic impacts on electrophysiology and network stability. Moreover, while specific PD mutations have been associated with distinct electrophysiological changes, a systematic analysis of genetic and epigenetic interactions across diverse models is still lacking. Comparative studies could illuminate common and divergent electrophysiological markers that serve as robust biomarkers for disease progression and treatment response, ultimately guiding the development of targeted interventions.

To assist in improving both the quantity and quality of information extracted from electrophysiology, as well as the multi-modal integration of data, artificial intelligence (AI) could come into play (Freestone et al., 2017; Ng et al., 2022). AI can streamline the analysis of complex *in vitro* recordings, such as patch-clamp and MEA data. Deep learning models, like the densely linked bidirectional long short-term memory model, which achieved 99.6% accuracy in PD classification using EEG data (Obayya et al., 2023), could be adapted to detect subtle neuronal firing patterns or synaptic dysfunction. AI can also identify PD-specific signatures in electrophysiological data, accelerating the characterization of disease phenotypes and advancing diagnostic and therapeutic strategies.

Beyond signal analysis, AI plays a crucial role in biomarker detection and multi-modal data integration (Siontis et al., 2021; Winchester et al., 2023). AI-driven models can combine electrophysiological, molecular, genetic, and imaging data, offering deeper mechanistic insights into PD pathophysiology (Gupta et al., 2023). Hybrid AI approaches, such as those integrating radiomics and deep learning, highlight the potential of merging diverse data streams for more accurate disease detection (Otero-Losada et al., 2025)*. In vitro*, AI can correlate electrophysiological abnormalities with αSyn aggregation, allowing for a systems-level understanding of disease progression.

Further complicating the landscape is the heterogeneity of cellular sources used in PD research (Ferrari et al., 2020). Immortalized cell lines, primary neurons, and hiPSCs all have advantages and limitations. While cell lines offer ease of culture and reproducibility (L. Gao et al., 2016; Lopes et al., 2017; Presgraves et al., 2003), they lack the genetic relevance of patient-derived cells. Primary neurons offer high physiological fidelity (Garcia-Munoz et al., 2015; Masuko et al., 1992; Weinert et al., 2015) but are limited by their variability and differences between murine and human phenotypes (Ferrari et al., 2020). In contrast, hiPSCs provide patient-specific genetic backgrounds and the ability to generate disease-relevant neuronal subtypes, making them a promising tool for personalized medicine (Nguyen et al., 2011; Sánchez-Danés et al., 2012; Soldner et al., 2011).

However, the variability in methodologies and conditions across different studies presents significant limitations, making it difficult to draw consistent conclusions and compare studies. The necessity for standardization is crucial, as it ensures the generation of more reliable, reproducible and comparable results (Kılıç and Yılmaz, 2024; Reyes et al., 2024). Standard protocols should be cost-effective, robust, consistently reproducible, and easy to implement to ensure broad applicability. By establishing universal experimental protocols, researchers can enhance scientific validity and accelerate the translation of *in vitro* findings into clinical applications. A possibility would be to develop standard protocols for electrode placement (in the case of patch-clamp studies), cell seeding density over the electrodes area (for MEAs recordings), stimulation parameters, and data acquisition. The implementation of standardized data processing and analysis methods is necessary to compare results across studies, as well as harmonizing cell and tissue models. Additionally, the use of open data repositories facilitates transparency and large-scale validation. These measures collectively promote the robustness and translational applicability of electrophysiological studies, not only in PD research.

Future studies should also focus on incorporating other cell types, such as astrocytes, microglia, and oligodendrocytes, into co-culture systems or organoids to capture the complex cellular interactions driving PD pathophysiology (Reumann et al., 2023). The contributions of neuroinflammation and glial-mediated synaptic modulation are likely critical in shaping electrophysiological outcomes (Hindeya Gebreyesus and Gebrehiwot Gebremichael, 2020; Wang et al., 2021). Moreover, even though organoid-based models have led to remarkable advancements, as previously discussed in this review, they are still rarely used for the electrophysiological study of PD.

In conclusion, *in vitro* models for electrophysiological studies have significantly advanced our understanding of PD by uncovering key disruptions in neuronal function and network dynamics. To fully realize their potential, future research must prioritize long-term electrophysiological studies, elucidate the mechanisms of pathological synchronization, and integrate multi-scale data, including insights from calcium imaging. Moreover, a systematic exploration of neuromodulatory influences, genetic and epigenetic interactions, and the contributions of non-neuronal cell types will be essential in developing more comprehensive and physiologically relevant models. These efforts will not only improve reproducibility but also facilitate the identification of robust electrophysiological biomarkers and accelerate the development of targeted therapeutic strategies for Parkinson’s disease.

## References

[ref1] Abdelrahman S. Alsanie W. F. Khan Z. N. Albalawi H. I. Felimban R. I. Moretti M.. (2022). A Parkinson’s disease model composed of 3D bioprinted dopaminergic neurons within a biomimetic peptide scaffold. Biofabrication 14:044103. doi: 10.1088/1758-5090/ac7eec, PMID: 35793642

[ref2] Abdullah R. Basak I. Patil K. S. Alves G. Larsen J. P. Møller S. G. (2015). Parkinson’s disease and age: the obvious but largely unexplored link. Exp. Gerontol. 68, 33–38. doi: 10.1016/j.exger.2014.09.014, PMID: 25261764

[ref3] Amin H. Nieus T. Lonardoni D. Maccione A. Berdondini L. (2017). High-resolution bioelectrical imaging of Aβ-induced network dysfunction on CMOS-MEAs for neurotoxicity and rescue studies. Sci. Rep. 7:2460. doi: 10.1038/s41598-017-02635-x, PMID: 28550283 PMC5446416

[ref4] Angela Cenci M. (2014). Presynaptic mechanisms of L-DOPA-induced dyskinesia: the findings, the debate, the therapeutic implications. Front. Neurol. 5:73–87. doi: 10.3389/fneur.2014.00242, PMID: 25566170 PMC4266027

[ref5] Armstrong M. J. Okun M. S. (2020). Diagnosis and treatment of Parkinson disease: a review. JAMA - J. American Medical Association 323, 548–560. doi: 10.1001/jama.2019.22360 32044947

[ref6] Atik A. Stewart T. Zhang J. (2016). Alpha-Synuclein as a biomarker for Parkinson’s disease. Brain Pathol. 26, 410–418. doi: 10.1111/bpa.12370, PMID: 26940058 PMC6245641

[ref7] Barré-Sinoussi F. Montagutelli X. (2015). Animal models are essential to biological research: issues and perspectives. Future Sci. OA 1:FSO63. doi: 10.4155/fso.15.63, PMID: 28031915 PMC5137861

[ref8] Beccano-Kelly D. A. Cherubini M. Mousba Y. Cramb K. M. L. Giussani S. Caiazza M. C.. (2023). Calcium dysregulation combined with mitochondrial failure and electrophysiological maturity converge in Parkinson’s iPSC-dopamine neurons. IScience 26:107044. doi: 10.1016/j.isci.2023.107044, PMID: 37426342 PMC10329047

[ref9] Bednarczyk P. Kampa R. P. Gałecka S. Sęk A. Walewska A. Koprowski P. (2021). Patch-clamp recording of the activity of ion channels in the inner mitochondrial membrane, 235–248. 10.1007/978-1-0716-1266-8_18 34060046

[ref10] Beudel M. Brown P. (2016). Adaptive deep brain stimulation in Parkinson’s disease. Parkinsonism Relat. Disord. 22, S123–S126. doi: 10.1016/j.parkreldis.2015.09.028, PMID: 26411502 PMC4671979

[ref11] Bloem B. R. Okun M. S. Klein C. (2021). Parkinson’s disease. Lancet 397, 2284–2303. doi: 10.1016/S0140-6736(21)00218-X 33848468

[ref12] Branch S. Y. Chen C. Sharma R. Lechleiter J. D. Li S. Beckstead M. J. (2016). Dopaminergic neurons exhibit an age-dependent decline in electrophysiological parameters in the MitoPark mouse model of Parkinson’s disease. J. Neurosci. 36, 4026–4037. doi: 10.1523/JNEUROSCI.1395-15.2016, PMID: 27053209 PMC4821912

[ref13] Cattaneo C. Jost W. H. (2023). Pain in Parkinson’s disease: pathophysiology, classification and treatment. J. Integr. Neurosci. 22:13–19. doi: 10.31083/j.jin2205132 37735139

[ref14] Chan C. S. Glajch K. E. Gertler T. S. Guzman J. N. Mercer J. N. Lewis A. S.. (2011). HCN channelopathy in external globus pallidus neurons in models of Parkinson’s disease. Nat. Neurosci. 14, 85–92. doi: 10.1038/nn.2692, PMID: 21076425 PMC3058391

[ref15] Chen R. Gu X. Wang X. (2022). α-Synuclein in Parkinson’s disease and advances in detection. Clin. Chim. Acta 529, 76–86. doi: 10.1016/j.cca.2022.02.006, PMID: 35176268

[ref16] Chen T. Yang Y. Fan Luo P. Liu W. Dai S. H. Zheng X. R.. (2013). Homer1 knockdown protects dopamine neurons through regulating calcium homeostasis in an in vitro model of Parkinson’s disease. Cell. Signal. 25, 2863–2870. doi: 10.1016/j.cellsig.2013.09.004, PMID: 24036210

[ref17] Cole R. C. Okine D. N. Yeager B. E. Narayanan N. S. (2022). Neuromodulation of cognition in Parkinson’s disease 269, 435–455. doi: 10.1016/bs.pbr.2022.01.016, PMID: 35248205 PMC9199111

[ref18] de Bie R. M. A. Clarke C. E. Espay A. J. Fox S. H. Lang A. E. (2020). Initiation of pharmacological therapy in Parkinson’s disease: when, why, and how. Lancet Neurol. 19, 452–461. doi: 10.1016/S1474-4422(20)30036-3 32171387

[ref19] Do Q. B. Noor H. Marquez-Gomez R. Cramb K. M. L. Ng B. Abbey A.. (2024). Early deficits in an in vitro striatal microcircuit model carrying the Parkinson’s GBA-N370S mutation. NPJ Parkinson’s Dis. 10:82. doi: 10.1038/s41531-024-00694-2, PMID: 38609392 PMC11014935

[ref20] Dong-Chen X. Yong C. Yang X. Chen-Yu S. Li-Hua P. (2023). Signaling pathways in Parkinson’s disease: molecular mechanisms and therapeutic interventions. Signal Transduct. Target. Ther. 8:73. doi: 10.1038/s41392-023-01353-3, PMID: 36810524 PMC9944326

[ref21] Fasano A. Aquino C. C. Krauss J. K. Honey C. R. Bloem B. R. (2015). Axial disability and deep brain stimulation in patients with Parkinson disease. Nat. Rev. Neurol. 11, 98–110. doi: 10.1038/nrneurol.2014.252 25582445

[ref22] Ferrari E. Cardinale A. Picconi B. Gardoni F. (2020). From cell lines to pluripotent stem cells for modelling Parkinson’s disease. J. Neurosci. Methods 340. doi: 10.1016/j.jneumeth.2020.108741 32311374

[ref23] Freestone D. R. Layton K. J. Kuhlmann L. Cook M. J. (2017). Statistical performance analysis of data-driven neural models. Int. J. Neural Syst. 27:1650045. doi: 10.1142/S0129065716500453, PMID: 27776437

[ref24] Gao C. Liu J. Tan Y. Chen S. (2020). Freezing of gait in Parkinson’s disease: pathophysiology, risk factors and treatments. Translational Neurodegen. 9:12. doi: 10.1186/s40035-020-00191-5, PMID: 32322387 PMC7161193

[ref25] Gao L. Zhou W. Symmes B. Freed C. R. (2016). Re-cloning the N27 dopamine cell line to improve a cell culture model of Parkinson’s disease. PLoS One 11:e0160847. doi: 10.1371/journal.pone.0160847, PMID: 27512998 PMC4981411

[ref26] Garcia-Munoz M. Taiiiefer E. Pnini R. Vickers C. Miller J. Arbuthnott G. W. (2015). Rebuilding a realistic corticostriatal “social network” from dissociated cells. Front. Syst. Neurosci. 9:63–71. doi: 10.3389/fnsys.2015.00063, PMID: 25941477 PMC4403293

[ref27] Ghovanloo M.-R. Tyagi S. Zhao P. Kiziltug E. Estacion M. Dib-Hajj S. D.. (2023). High-throughput combined voltage-clamp/current-clamp analysis of freshly isolated neurons. Cell Reports Methods 3:100385. doi: 10.1016/j.crmeth.2022.100385, PMID: 36814833 PMC9939380

[ref28] Gupta R. Kumari S. Senapati A. Ambasta R. K. Kumar P. (2023). New era of artificial intelligence and machine learning-based detection, diagnosis, and therapeutics in Parkinson’s disease. Ageing Res. Rev. 90:102013. doi: 10.1016/j.arr.2023.102013, PMID: 37429545

[ref29] Hamill O. P. Marty A. Neher E. Sakmann B. Sigworth F. J. (1981). Improved patch-clamp techniques for high-resolution current recording from cells and cell-free membrane patches. Pflugers Arch. - Eur. J. Physiol. 391, 85–100. doi: 10.1007/BF00656997 6270629

[ref30] Hariz M. Blomstedt P. (2022). Deep brain stimulation for Parkinson’s disease. J. Intern. Med. 292, 292, 764–778. doi: 10.1111/joim.13541, PMID: 35798568 PMC9796446

[ref31] Hayes M. T. (2019). Parkinson’s disease and parkinsonism. Am. J. Med. 132, 802–807. doi: 10.1016/j.amjmed.2019.03.001, PMID: 30890425

[ref32] Hill C. L. Stephens G. J. (2021). An introduction to patch clamp recording, 2188:1–19. doi: 10.1007/978-1-0716-0818-0_1 33119844

[ref33] Hindeya Gebreyesus H. Gebrehiwot Gebremichael T. (2020). The potential role of astrocytes in Parkinson’s disease (PD). Medical Sci. MDPI, Medical Sciences 8. doi: 10.3390/medsci8010007, PMID: 32012713 PMC7151567

[ref34] Hou Y. Dan X. Babbar M. Wei Y. Hasselbalch S. G. Croteau D. L.. (2019). Ageing as a risk factor for neurodegenerative disease. Nat. Rev. Neurol. 15, 565–581. doi: 10.1038/s41582-019-0244-7 31501588

[ref35] Jäckel D. Bakkum D. J. Russell T. L. Müller J. Radivojevic M. Frey U.. (2017). Combination of high-density microelectrode Array and patch clamp recordings to enable studies of multisynaptic integration. Sci. Rep. 7:978. doi: 10.1038/s41598-017-00981-4, PMID: 28428560 PMC5430511

[ref36] Jankovic J. (2008). Parkinson’s disease: clinical features and diagnosis. J. Neurol. Neurosurg. Psychiatry 79, 368–376. doi: 10.1136/jnnp.2007.131045, PMID: 18344392

[ref37] Jankovic J. Tan E. K. (2020). Parkinson’s disease: Etiopathogenesis and treatment. J. Neurol. Neurosurg. Psychiatry 91, 795–808. doi: 10.1136/jnnp-2019-322338, PMID: 32576618

[ref38] Kamath T. Abdulraouf A. Burris S. J. Langlieb J. Gazestani V. Nadaf N. M.. (2022). Single-cell genomic profiling of human dopamine neurons identifies a population that selectively degenerates in Parkinson’s disease. Nat. Neurosci. 25, 588–595. doi: 10.1038/s41593-022-01061-1 35513515 PMC9076534

[ref39] Kılıç K. D. Yılmaz Z. S. (2024). Importance of in vitro embryo model procedure standardization. J. Clin. Lab. Anal. 38:e25082. doi: 10.1002/jcla.25082, PMID: 39072781 PMC11317767

[ref40] Kouroupi G. Antoniou N. Prodromidou K. Taoufik E. Matsas R. (2020). Patient-derived induced pluripotent stem cell-based models in Parkinson’s disease for drug identification. Int. J. Mol. Sci. 21:7113. doi: 10.3390/ijms21197113, PMID: 32993172 PMC7582359

[ref41] Kouroupi G. Taoufik E. Vlachos I. S. Tsioras K. Antoniou N. Papastefanaki F.. (2017). Defective synaptic connectivity and axonal neuropathology in a human iPSC-based model of familial Parkinson’s disease. Proc. Natl. Acad. Sci. USA 114, E3679–E3688. doi: 10.1073/pnas.1617259114, PMID: 28416701 PMC5422768

[ref42] Laperle A. H. Sances S. Yucer N. Dardov V. J. Garcia V. J. Ho R.. (2020). iPSC modeling of young-onset Parkinson’s disease reveals a molecular signature of disease and novel therapeutic candidates. Nat. Med. 26, 289–299. doi: 10.1038/s41591-019-0739-1 31988461

[ref43] Lin L. Göke J. Cukuroglu E. Dranias M. R. VanDongen A. M. J. Stanton L. W. (2016). Molecular features underlying neurodegeneration identified through in vitro modeling of genetically diverse Parkinson’s disease patients. Cell Rep. 15, 2411–2426. doi: 10.1016/j.celrep.2016.05.022, PMID: 27264186

[ref44] Lopes F. M. Bristot I. J. da Motta L. L. Parsons R. B. Klamt F. (2017). Mimicking Parkinson’s disease in a dish: merits and pitfalls of the Most commonly used dopaminergic in vitro models. NeuroMolecular Med. 19, 241–255. doi: 10.1007/s12017-017-8454-x, PMID: 28721669

[ref45] Marogianni C. Sokratous M. Dardiotis E. Hadjigeorgiou G. M. Bogdanos D. Xiromerisiou G. (2020). Neurodegeneration and inflammation—an interesting interplay in Parkinson’s disease. Int. J. Mol. Sci. 21:8421. doi: 10.3390/ijms21228421, PMID: 33182554 PMC7697354

[ref46] Masuko S. Nakajima S. Nakajima Y. (1992). Dissociated high-purity dopaminergic neuron cultures from the substantia nigra and the ventral tegmental area of the postnatal rat. Neuroscience 49, 347–364. doi: 10.1016/0306-4522(92)90101-7, PMID: 1359454

[ref47] Monchy N. Modolo J. Houvenaghel J.-F. Voytek B. Duprez J. (2024). Changes in electrophysiological aperiodic activity during cognitive control in Parkinson’s disease. Brain Commun. 6:306–324. doi: 10.1093/braincomms/fcae306, PMID: 39301291 PMC11411214

[ref48] Morris H. R. Spillantini M. G. Sue C. M. Williams-Gray C. H. (2024). The pathogenesis of Parkinson’s disease. Lancet 403, 293–304. doi: 10.1016/S0140-6736(23)01478-2 38245249

[ref49] Murakami H. Shiraishi T. Umehara T. Omoto S. Iguchi Y. (2023). Recent advances in drug therapy for Parkinson’s disease. Intern. Med. 62, 33–42. doi: 10.2169/internalmedicine.8940-21, PMID: 35110492 PMC9876715

[ref50] Nakamura R. Nonaka R. Oyama G. Jo T. Kamo H. Nuermaimaiti M.. (2023). A defined method for differentiating human iPSCs into midbrain dopaminergic progenitors that safely restore motor deficits in Parkinson’s disease. Front. Neurosci. 17. doi: 10.3389/fnins.2023.1202027, PMID: 37502682 PMC10368972

[ref51] Negri J. Menon V. Young-Pearse T. L. (2020). Assessment of spontaneous neuronal activity in vitro using multi-well multi-electrode arrays: implications for assay development. ENeuro 7, ENEURO.0080–ENEU19.2019. doi: 10.1523/ENEURO.0080-19.2019, PMID: 31896559 PMC6984810

[ref52] Ng B. Nayyar S. Chauhan V. S. (2022). The role of artificial intelligence and machine learning in clinical cardiac electrophysiology. Can. J. Cardiol. 38, 246–258. doi: 10.1016/j.cjca.2021.07.016, PMID: 34333029

[ref53] Nguyen H. N. Byers B. Cord B. Shcheglovitov A. Byrne J. Gujar P.. (2011). LRRK2 mutant iPSC-derived da neurons demonstrate increased susceptibility to oxidative stress. Cell Stem Cell 8, 267–280. doi: 10.1016/j.stem.2011.01.013, PMID: 21362567 PMC3578553

[ref54] O’Connell A. Quinlan L. Kwakowsky A. (2024). β-amyloid’s neurotoxic mechanisms as defined by in vitro microelectrode arrays: a review. Pharmacol. Res. 209:107436. doi: 10.1016/j.phrs.2024.107436, PMID: 39369863

[ref55] Obayya M. Saeed M. K. Maashi M. Alotaibi S. S. Salama A. S. Ahmed Hamza M. (2023). A novel automated Parkinson’s disease identification approach using deep learning and EEG. PeerJ Computer Sci. 9:e1663. doi: 10.7717/peerj-cs.1663, PMID: 38077610 PMC10703017

[ref56] Obien M. E. J. Deligkaris K. Bullmann T. Bakkum D. J. Frey U. (2015). Revealing neuronal function through microelectrode array recordings. Front. Neurosci. 8:423. doi: 10.3389/fnins.2014.00423, PMID: 25610364 PMC4285113

[ref57] Olanow C. W. Obeso J. A. Stocchi F. (2006). Continuous dopamine-receptor treatment of Parkinson’s disease: scientific rationale and clinical implications. Lancet Neurol. 5, 677–687. doi: 10.1016/S1474-4422(06)70521-X, PMID: 16857573

[ref58] Otero-Losada M. Perez Lloret S. Capani F. Falup-Pecurariu C. (2025). Editorial: contribution of artificial intelligence-based tools to the study of Parkinson’s disease and other movement disorders. Front. Aging Neurosci. 17:1567706. doi: 10.3389/fnagi.2025.1567706, PMID: 40103925 PMC11914123

[ref59] Ozgun A. Lomboni D. J. Aylsworth A. Macdonald A. Staines W. A. Martina M.. (2024). Unraveling the assembloid: real-time monitoring of dopaminergic neurites in an inter-organoid pathway connecting midbrain and striatal regions. Materials Today Bio 25:100992. doi: 10.1016/j.mtbio.2024.100992, PMID: 38371467 PMC10873721

[ref60] Pirker W. Katzenschlager R. Hallett M. Poewe W. (2023). Pharmacological treatment of tremor in Parkinson’s disease revisited. J. Parkinsons Dis. 13, 127–144. doi: 10.3233/JPD-225060, PMID: 36847017 PMC10041452

[ref61] Poewe W. Seppi K. Tanner C. M. Halliday G. M. Brundin P. Volkmann J.. (2017). Parkinson disease. Nat. Rev. Dis. Prim. 3, 1–21. doi: 10.1038/nrdp.2017.13 28332488

[ref62] Presgraves S. P. Ahmed T. Borwege S. Joyce J. N. (2003). Terminally differentiated SH-SY5Y cells provide a model system for studying neuroprotective effects of dopamine agonists. Neurotox. Res. 5, 579–598. doi: 10.1007/BF03033178, PMID: 15111235

[ref63] Puopolo M. Raviola E. Bean B. P. (2007). Roles of subthreshold calcium current and sodium current in spontaneous firing of mouse midbrain dopamine neurons. J. Neurosci. 27, 645–656. doi: 10.1523/JNEUROSCI.4341-06.2007, PMID: 17234596 PMC6672803

[ref64] Rakovic A. Voß D. Vulinovic F. Meier B. Hellberg A.-K. Nau C.. (2022). Electrophysiological properties of induced pluripotent stem cell-derived midbrain dopaminergic neurons correlate with expression of tyrosine hydroxylase. Front. Cell. Neurosci. 16:24–38. doi: 10.3389/fncel.2022.817198, PMID: 35401116 PMC8983830

[ref65] Rassoulou F. Steina A. Hartmann C. J. Vesper J. Butz M. Schnitzler A.. (2024). Exploring the electrophysiology of Parkinson’s disease with magnetoencephalography and deep brain recordings. Scientific Data 11:889. doi: 10.1038/s41597-024-03768-1, PMID: 39147788 PMC11327342

[ref66] Reumann D. Krauditsch C. Novatchkova M. Sozzi E. Wong S. N. Zabolocki M.. (2023). In vitro modeling of the human dopaminergic system using spatially arranged ventral midbrain–striatum–cortex assembloids. Nat. Methods 20, 2034–2047. doi: 10.1038/s41592-023-02080-x, PMID: 38052989 PMC10703680

[ref67] Reyes D. R. Esch M. B. Ewart L. Nasiri R. Herland A. Sung K.. (2024). From animal testing to in vitro systems: advancing standardization in microphysiological systems. Lab Chip 24, 1076–1087. doi: 10.1039/D3LC00994G, PMID: 38372151

[ref68] Rocha E. M. De Miranda B. Sanders L. H. (2018). Alpha-synuclein: pathology, mitochondrial dysfunction and neuroinflammation in Parkinson’s disease. Neurobiol. Dis. 109, 249–257. doi: 10.1016/j.nbd.2017.04.004, PMID: 28400134

[ref69] Ronchi S. Buccino A. P. Prack G. Kumar S. S. Schröter M. Fiscella M.. (2021). Electrophysiological phenotype characterization of human iPSC-derived neuronal cell lines by means of high-density microelectrode arrays. Advan. Biol. 5:1–16. doi: 10.1002/adbi.202000223, PMID: 33729694 PMC7610355

[ref70] Rosholm K. R. Boddum K. Lindquist A. (2021). Perforated whole-cell recordings in automated patch clamp electrophysiology, 93–108. 10.1007/978-1-0716-0818-0_5 33119848

[ref71] Ross A. Xing V. Wang T. T. Bureau S. C. Link G. A. Fortin T.. (2020). Alleviating toxic α-Synuclein accumulation by membrane depolarization: evidence from an in vitro model of Parkinson’s disease. Mol. Brain 13:108–119. doi: 10.1186/s13041-020-00648-8, PMID: 32736645 PMC7395353

[ref72] Sánchez-Danés A. Richaud-Patin Y. Carballo-Carbajal I. Jiménez-Delgado S. Caig C. Mora S.. (2012). Disease-specific phenotypes in dopamine neurons from human iPS-based models of genetic and sporadic Parkinson’s disease. EMBO Mol. Med. 4, 380–395. doi: 10.1002/emmm.201200215, PMID: 22407749 PMC3403296

[ref73] Schindler A. Pizzorni N. Cereda E. Cosentino G. Avenali M. Montomoli C.. (2021). Consensus on the treatment of dysphagia in Parkinson’s disease. J. Neurol. Sci. 430:008. doi: 10.1016/j.jns.2021.120008 34624796

[ref74] Segev A. Garcia-Oscos F. Kourrich S. (2016). Whole-cell patch-clamp recordings in brain slices. J. Vis. Exp. 112:e54024. doi: 10.3791/54024, PMID: 27341060 PMC4927800

[ref75] Siontis K. C. Noseworthy P. A. Attia Z. I. Friedman P. A. (2021). Artificial intelligence-enhanced electrocardiography in cardiovascular disease management. Nat. Rev. Cardiol. 18, 465–478. doi: 10.1038/s41569-020-00503-2, PMID: 33526938 PMC7848866

[ref76] Smits L. M. Reinhardt L. Reinhardt P. Glatza M. Monzel A. S. Stanslowsky N.. (2019). Modeling Parkinson’s disease in midbrain-like organoids. NPJ Parkinson’s Dis. 5:5. doi: 10.1038/s41531-019-0078-4, PMID: 30963107 PMC6450999

[ref77] Soldner F. Laganière J. Cheng A. W. Hockemeyer D. Gao Q. Alagappan R.. (2011). Generation of isogenic pluripotent stem cells differing exclusively at two early onset parkinson point mutations. Cell 146, 318–331. doi: 10.1016/j.cell.2011.06.019, PMID: 21757228 PMC3155290

[ref78] Spira M. E. Hai A. (2013). Multi-electrode array technologies for neuroscience and cardiology. Nat. Nanotechnol. 8, 83–94. doi: 10.1038/nnano.2012.265 23380931

[ref79] Sturchio A. Rocha E. M. Kauffman M. A. Marsili L. Mahajan A. Saraf A. A.. (2024). Recalibrating the why and whom of animal models in Parkinson disease: a Clinician’s perspective. Brain Sci. 14:151–169. doi: 10.3390/brainsci14020151, PMID: 38391726 PMC10887152

[ref80] Takasuna K. Asakura K. Araki S. Ando H. Kazusa K. Kitaguchi T.. (2017). Comprehensive in vitro cardiac safety assessment using human stem cell technology: overview of CSAHi HEART initiative. J. Pharmacol. Toxicol. Methods 83, 42–54. doi: 10.1016/j.vascn.2016.09.004, PMID: 27646297

[ref81] Ting J. T. Daigle T. L. Chen Q. Feng G. (2014). Acute brain slice methods for adult and aging animals: application of targeted patch clamp analysis and Optogenetics, 1183:221–242. doi: 10.1007/978-1-4939-1096-0_14 PMC4219416 25023312

[ref82] Toh M. F. Brooks J. M. Strassmaier T. Haedo R. J. Puryear C. B. Roth B. L.. (2020). Application of high-throughput automated patch-clamp electrophysiology to study voltage-gated Ion Channel function in primary cortical cultures. SLAS Discovery 25, 447–457. doi: 10.1177/2472555220902388, PMID: 32003306

[ref83] Tønnesen J. Parish C. L. Sørensen A. T. Andersson A. Lundberg C. Deisseroth K.. (2011). Functional integration of grafted neural stem cell-derived dopaminergic neurons monitored by optogenetics in an in vitro Parkinson model. PLoS One 6:e17560. doi: 10.1371/journal.pone.0017560, PMID: 21394212 PMC3048875

[ref84] Travagli R. A. Browning K. N. Camilleri M. (2020). Parkinson disease and the gut: new insights into pathogenesis and clinical relevance. Nat. Rev. Gastroenterol. Hepatol. 17, 673–685. doi: 10.1038/s41575-020-0339-z 32737460

[ref85] Tysnes O. B. Storstein A. (2017). Epidemiology of Parkinson’s disease. J. Neural Transm. 124, 901–905. doi: 10.1007/s00702-017-1686-y 28150045

[ref86] Valderhaug V. D. Heiney K. Ramstad O. H. Bråthen G. Kuan W. L. Nichele S.. (2021). Early functional changes associated with alpha-synuclein proteinopathy in engineered human neural networks. Am. J. Phys. Cell Phys. 320, C1141–C1152. doi: 10.1152/AJPCELL.00413.2020, PMID: 33950697

[ref87] Vargas J. Y. Grudina C. Zurzolo C. (2019). The prion-like spreading of α-synuclein: from in vitro to in vivo models of Parkinson’s disease. Ageing Res. Rev. 50:89:101. doi: 10.1016/j.arr.2019.01.012 30690184

[ref88] Viegas M. P. C. Santos L. E. C. Aarão M. C. Cecilio S. G. Medrado J. M. Pires A. C.. (2023). The nonsynaptic plasticity in Parkinson’s disease: insights from an animal model. Clinics 78:100242. doi: 10.1016/j.clinsp.2023.100242, PMID: 37480642 PMC10387572

[ref89] Virdi G. S. Choi M. L. Evans J. R. Yao Z. Athauda D. Strohbuecker S.. (2022). Protein aggregation and calcium dysregulation are hallmarks of familial Parkinson’s disease in midbrain dopaminergic neurons. NPJ Parkinson’s Dis. 8:162. doi: 10.1038/s41531-022-00423-7, PMID: 36424392 PMC9691718

[ref90] Voges J. Hilker R. Bötzel K. Kiening K. L. Kloss M. Kupsch A.. (2007). Thirty days complication rate following surgery performed for deep-brain-stimulation. Mov. Disord. 22, 1486–1489. doi: 10.1002/mds.21481, PMID: 17516483

[ref91] Volkmann J. Daniels C. Witt K. (2010). Neuropsychiatric effects of subthalamic neurostimulation in Parkinson disease. Nature Rev. Neurology 6, 487–498. doi: 10.1038/nrneurol.2010.111, PMID: 20680036

[ref92] Wang C. Yang T. Liang M. Xie J. Song N. (2021). Astrocyte dysfunction in Parkinson’s disease: from the perspectives of transmitted α-synuclein and genetic modulation. Translational Neurodegen. 10:39. doi: 10.1186/s40035-021-00265-y, PMID: 34657636 PMC8522040

[ref93] Weinert M. Selvakumar T. Tierney T. S. Alavian K. N. (2015). Isolation, culture and long-term maintenance of primary mesencephalic dopaminergic neurons from embryonic rodent brains. J. Vis. Exp. 96:96–101. doi: 10.3791/52475, PMID: 25741798 PMC4354654

[ref94] Winchester L. M. Harshfield E. L. Shi L. Badhwar A. (2023). Artificial intelligence for biomarker discovery in Alzheimer’s disease and dementia. Alzheimers Dement. 19, 5860–5871. doi: 10.1002/alz.13390, PMID: 37654029 PMC10840606

[ref95] Woodard C. M. Campos B. A. Kuo S. H. Nirenberg M. J. Nestor M. W. Zimmer M.. (2014). IPSC-derived dopamine neurons reveal differences between monozygotic twins discordant for parkinson’s disease. Cell Rep. 9, 1173–1182. doi: 10.1016/j.celrep.2014.10.023, PMID: 25456120 PMC4255586

[ref96] Wulansari N. Darsono W. H. W. Woo H.-J. Chang M.-Y. Kim J. Bae E.-J.. (2021). Neurodevelopmental defects and neurodegenerative phenotypes in human brain organoids carrying Parkinson’s disease-linked DNAJC6 mutations. Science. Advances 7. doi: 10.1126/sciadv.abb1540, PMID: 33597231 PMC7888924

[ref97] Xu W. Wang J. Li X. N. Liang J. Song L. Wu Y.. (2023). Neuronal and synaptic adaptations underlying the benefits of deep brain stimulation for Parkinson’s disease. Translational Neurodegen. Translational Neurodegeneration, BMC, Springer Nature 12:55. doi: 10.1186/s40035-023-00390-w, PMID: 38037124 PMC10688037

[ref98] Zeiss C. J. Allore H. G. Beck A. P. (2017). Established patterns of animal study design undermine translation of disease-modifying therapies for Parkinson’s disease. PLoS One 12:e0171790. doi: 10.1371/journal.pone.0171790, PMID: 28182759 PMC5300282

[ref99] Zheng X. Han D. Liu W. Wang X. Pan N. Wang Y.. (2023). Human iPSC-derived midbrain organoids functionally integrate into striatum circuits and restore motor function in a mouse model of Parkinson’s disease. Theranostics 13, 2673–2692. doi: 10.7150/thno.80271, PMID: 37215566 PMC10196819

[ref100] Zhu Z.-T. Shen K.-Z. Johnson S. W. (2002). Pharmacological identification of inward current evoked by dopamine in rat subthalamic neurons in vitro. Neuropharmacology 42, 772–781. doi: 10.1016/S0028-3908(02)00035-7, PMID: 12015203

[ref101] Zygogianni O. Antoniou N. Kalomoiri M. Kouroupi G. Taoufik E. Matsas R. (2019). In vivo phenotyping of familial Parkinson’s disease with human induced pluripotent stem cells: a proof-of-concept study. Neurochem. Res. 44, 1475–1493. doi: 10.1007/s11064-019-02781-w, PMID: 30989481

